# Alterations of cAMP-dependent signaling in dystrophic skeletal muscle

**DOI:** 10.3389/fphys.2013.00290

**Published:** 2013-10-17

**Authors:** Rüdiger Rudolf, Muzamil M. Khan, Danilo Lustrino, Siegfried Labeit, Ísis C. Kettelhut, Luiz C. C. Navegantes

**Affiliations:** ^1^Institute of Molecular and Cell Biology, University of Applied Sciences Mannheim, Mannheim, Germany; ^2^Institute of Toxicology and Genetics, Karlsruhe Institute of Technology, Eggenstein-Leopoldshafen, Germany; ^3^Departments of Physiology and Biochemistry and Immunology, School of Medicine of Ribeirão Preto, University of São Paulo, Ribeirao Preto, Brazil; ^4^Department of Integrative Pathophysiology, Universitätsmedizin Mannheim, Mannheim, Germany

**Keywords:** adrenoceptors, AKAP, endplate, dystrophy, PKA, metabolism, neuromuscular junction, skeletal muscle

## Abstract

Autonomic regulation processes in striated muscles are largely mediated by cAMP/PKA-signaling. In order to achieve specificity of signaling its spatial-temporal compartmentation plays a critical role. We discuss here how specificity of cAMP/PKA-signaling can be achieved in skeletal muscle by spatio-temporal compartmentation. While a microdomain containing PKA type I in the region of the neuromuscular junction (NMJ) is important for postsynaptic, activity-dependent stabilization of the nicotinic acetylcholine receptor (AChR), PKA type I and II microdomains in the sarcomeric part of skeletal muscle are likely to play different roles, including the regulation of muscle homeostasis. These microdomains are due to specific A-kinase anchoring proteins, like rapsyn and myospryn. Importantly, recent evidence indicates that compartmentation of the cAMP/PKA-dependent signaling pathway and pharmacological activation of cAMP production are aberrant in different skeletal muscles disorders. Thus, we discuss here their potential as targets for palliative treatment of certain forms of dystrophy and myasthenia. Under physiological conditions, the neuropeptide, α-calcitonin-related peptide, as well as catecholamines are the most-mentioned natural triggers for activating cAMP/PKA signaling in skeletal muscle. While the precise domains and functions of these first messengers are still under investigation, agonists of β_2_-adrenoceptors clearly exhibit anabolic activity under normal conditions and reduce protein degradation during atrophic periods. Past and recent studies suggest direct sympathetic innervation of skeletal muscle fibers. In summary, the organization and roles of cAMP-dependent signaling in skeletal muscle are increasingly understood, revealing crucial functions in processes like nerve-muscle interaction and muscle trophicity.

## Introduction

A variety of hormones and other first messengers employ cAMP-dependent signal transduction to exert their effects (Beavo and Brunton, 2002). Sympathetic activation of adrenergic receptors (or adrenoceptors) by catecholamines is the classical paradigm in this context. In skeletal muscle, catecholamines regulate many physiological functions, including force production (Oliver and Schäfer, 1895; Arreola et al., 1987; Cairns and Dulhunty, 1993a,b; Decostre et al., 2000), blood flow (Marshall, 1982; Saltin et al., 1998; Joyner and Casey, 2009), and metabolism (Gross et al., 1976; Navegantes et al., 2000, 2002). These effects might be mediated through endocrine delivery of epinephrine from the adrenal medulla, but adrenergic nerve terminals make also close contact with striated muscle fibers (Barker and Saito, 1981; Tadaki et al., 1995), suggesting direct release of norepinephrine onto muscle fibers in a neurotransmitter-like or paracrine fashion as it occurs at the heart (Zaglia et al., 2013). However, these aspects of adrenergic signaling on skeletal muscle are far from being established and are currently under investigation (see also below).

In skeletal muscle, catecholamines stimulate primarily β_2_-adrenergic receptors (β_2_-ARs). These are G protein-coupled receptors (GPCRs), which mostly couple to Gα_s_ and thus activate adenylyl cyclase (AC) (Liggett and Raymond, 1993), leading to an increase in cAMP levels, activation of cAMP-dependent protein kinase (PKA) and cAMP response element-binding protein (CREB) (Beavo and Brunton, 2002; Altarejos and Montminy, 2011). In parallel, cAMP signals through the “exchange protein activated directly by cAMP” (Epac) (Bos, 2003), and it regulates cyclic-nucleotide gated (CNGs) channels (Beavo and Brunton, 2002). The attenuation of cAMP effects is coordinated by the activation of cyclic nucleotide phosphodiesterases (PDEs), which are classified into 11 major families (PDE1-11) (Bloom, 2002; Omori and Kotera, 2007). In skeletal muscle, PDE4 appears to contribute to the majority of cAMP hydrolysis, accounting for more than 80% of the total PDE activity in this tissue (Bloom, 2002). Notably, a couple of different cAMP-regulating GPCRs are typically co-expressed in one and the same cell raising the evident issue of how the small inconspicuous molecule, cAMP, can trigger specific responses upon activation of a certain GPCR. This is also true for striated muscle where a plethora of physiological functions are subject to cAMP-dependent signaling. For skeletal muscle, Berdeaux and Stewart have recently reviewed the different functions of this pathway very nicely (Berdeaux and Stewart, 2012). Furthermore, lists of GPCRs (albeit likely not complete) expressed in heart and skeletal muscles can be found in reviews from Tang (Tang and Insel, 2004) and Jean-Baptiste (Jean-Baptiste et al., 2005), respectively. So, how is specificity gained in cAMP-dependent signaling pathways? The strongest current line of evidence supports a microdomain hypothesis, wherein spatial and temporal segregation of local rises of cAMP plus scaffolding of essential downstream effectors and targets of cAMP play pivotal roles (Steinberg and Brunton, 2001; Zaccolo et al., 2002; Zaccolo, 2011; Edwards et al., 2012). Central players in this scenario are variability of PKA isoforms, A kinase-anchoring proteins (AKAPs), and PDEs. In its inactive state, PKA is comprised of four subunits, i.e., two regulatory (PKA-R) and two catalytic subunits (PKA-C) (Taylor et al., 2008). Upon binding of cAMP to regulatory subunits, catalytic subunits are activated and detach from regulatory subunits in order to phosphorylate targets. In mammals, four isoforms of PKA-R are present, named as type Iα, Iβ, IIα, IIβ. While PDEs impair cAMP from spreading all over the cell through hydrolysis of the second messenger (Conti and Beavo, 2007; Francis et al., 2011), AKAPs serve as scaffolds integrating and anchoring many relevant partners of a GPCR-linked signaling pathway (Scott et al., 2013). Indeed, AKAPs not only bind to PKA (hence their name) but often also to GPCRs, ACs, PDEs, protein phosphatases, and target molecules (Edwards et al., 2012). Thereby, they integrate entire signaling complexes and guarantee high efficiency and fidelity of signal transduction. AKAPs belong to a large heterogeneous group of proteins, which do not share sequence homology but a set of functional properties. They typically exhibit a subcellular targeting domain, interaction domains with other components of signal pathways, and an amphipathic α-helical domain that serves as interaction terminal with PKA-R (Scott et al., 2013). Indeed, the PKA-R expose an N-terminal stretch called dimerization/docking- (D/D-) domain that combines with the AKAP α-helical parts at varying intensities and specificities. A typical and widely used means to test the functional impact of AKAP-PKA interaction is by introducing “AKAP disruptor peptides” which mimic the AKAP interaction domain and thereby release PKA-R from its normal microdomain (Scott et al., 2013). In summary, a large part of cAMP-dependent signaling specificity appears to arise from the interplay between PDEs and AKAPs and is sometimes subsumed under the term “PKA microdomain hypothesis,” recently described in depth in a couple of excellent reviews (Zaccolo, 2011; Edwards et al., 2012; Scott et al., 2013). The present contribution first addresses, how the “PKA microdomain hypothesis” applies to skeletal muscle and what are potential links to skeletal muscle diseases. In a second part, we review the current knowledge on how catecholamines regulate muscle trophicity.

## PKA microdomains in skeletal muscle

Investigations dealing with the PKA microdomain hypothesis usually address differential distribution patterns of distinct PKA-R isoforms. Owing to its highly regular striated patterning of sarcomeres, the contractile units of striated muscle, this tissue is particularly amenable to investigating the distribution of PKA-R isoforms relative to sarcomeric marker proteins. Another region of interest is the nerve-muscle synapse, termed as endplate or neuromuscular junction (NMJ), which instructs the rest of the muscle fiber to contract upon stimulation. For both parts there is now information concerning PKA-R distribution, alterations in diseased muscle, as well as causes underlying and consequences of these alterations. In the following we will describe the current state of knowledge regarding these points.

### Subsynaptic PKA microdomains

The NMJ is the synapse between motoneuron and muscle fiber and as such exerts the control over skeletal muscle contraction. The latter is triggered upon release of the neurotransmitter, acetylcholine, which activates postsynaptic nicotinic acetylcholine receptors (AChR) leading to an endplate potential and ultimately to muscle contraction. Notably, AChR reaches extremely high densities at the postsynaptic membrane of about 10,000 molecules per square micron and under normal conditions AChRs are metabolically very stable with a half-life of about 13 days (Fambrough, 1979). Principal functions attributed to cAMP/PKA-dependent signaling at the NMJ are synapse stabilization and the metabolic control of AChR stability and function (Li et al., 2001; Lanuza et al., 2002; Li et al., 2002; Nelson et al., 2003). *In situ* hybridization showed a peculiar accumulation of PKA-RIα transcripts in the NMJ region (Imaizumi-Scherrer et al., 1996) and immunohistochemical analyses found both, PKA-RIα and PKA-RIIα to be enriched close to the postsynaptic membrane (Perkins et al., 2001). However, different studies using fusions of different PKA-R D/D-domains with fluorescent proteins only revealed PKA-RIα but not PKA-RIIα in numerous punctiform structures just beneath the postsynaptic membrane (Barradeau et al., 2001, 2002; Röder et al., 2010; Choi et al., 2012). What do these puncta represent? The involvement of PKA signaling in AChR stabilization suggested them to be intracellular AChR carriers. As bona fide transmembrane proteins, the subunits of the pentameric AChR are generated and assembled in the endoplasmic reticulum, from where they are routed over the Golgi apparatus to the plasma membrane (Marchand et al., 2000, 2002; Marchand and Cartaud, 2002; Wanamaker and Green, 2005, 2007). Using different elegant labeling approaches with the highly AChR-selective snake venom, α-bungarotoxin, Engel et al. showed by electron microscopy that AChR is endocytosed in membrane-bound carriers (Engel et al., 1977; Fumagalli et al., 1982) and several groups established an activity-dependent metabolic stabilization of AChR (Fambrough, 1979; Levitt et al., 1980; Loring and Salpeter, 1980; Levitt and Salpeter, 1981; Stanley and Drachman, 1981, 1983; Salpeter and Loring, 1985; Shyng et al., 1991; Xu and Salpeter, 1997, 1999). Next, Akaaboune et al. demonstrated that AChR is recycled to the postsynaptic membrane in an activity-dependent manner (Akaaboune et al., 1999; Bruneau et al., 2005; Bruneau and Akaaboune, 2006). At this point a large part of the lifecycle of AChRs was described phenomenologically. However, amongst other open questions it remained unclear, what molecules underlie the regulatory decision-making (e.g., dwell at postsynaptic membrane vs. endocytose; recycle vs. degrade) and which machinery would support these processes. Most knowledge was gathered regarding the clustering of AChRs at the membrane, which is mediated by the release of neuronal agrin (Nitkin et al., 1987), binding of agrin to the MuSK co-receptor, LRP4, and activation of the receptor-tyrosine kinase MuSK (Kim et al., 2008; Zhang et al., 2008; Zong et al., 2012; Burden et al., 2013; Hubbard and Gnanasambandan, 2013). This then triggers AChR clustering by a yet ill-defined mechanism, which involves the receptor-associated protein of the synapse, rapsyn (Gillespie et al., 1996; Apel et al., 1997; Glass and Yancopoulos, 1997; Ruegg and Bixby, 1998; Fuhrer et al., 1999; Gautam et al., 1999).

**Figure 1:**
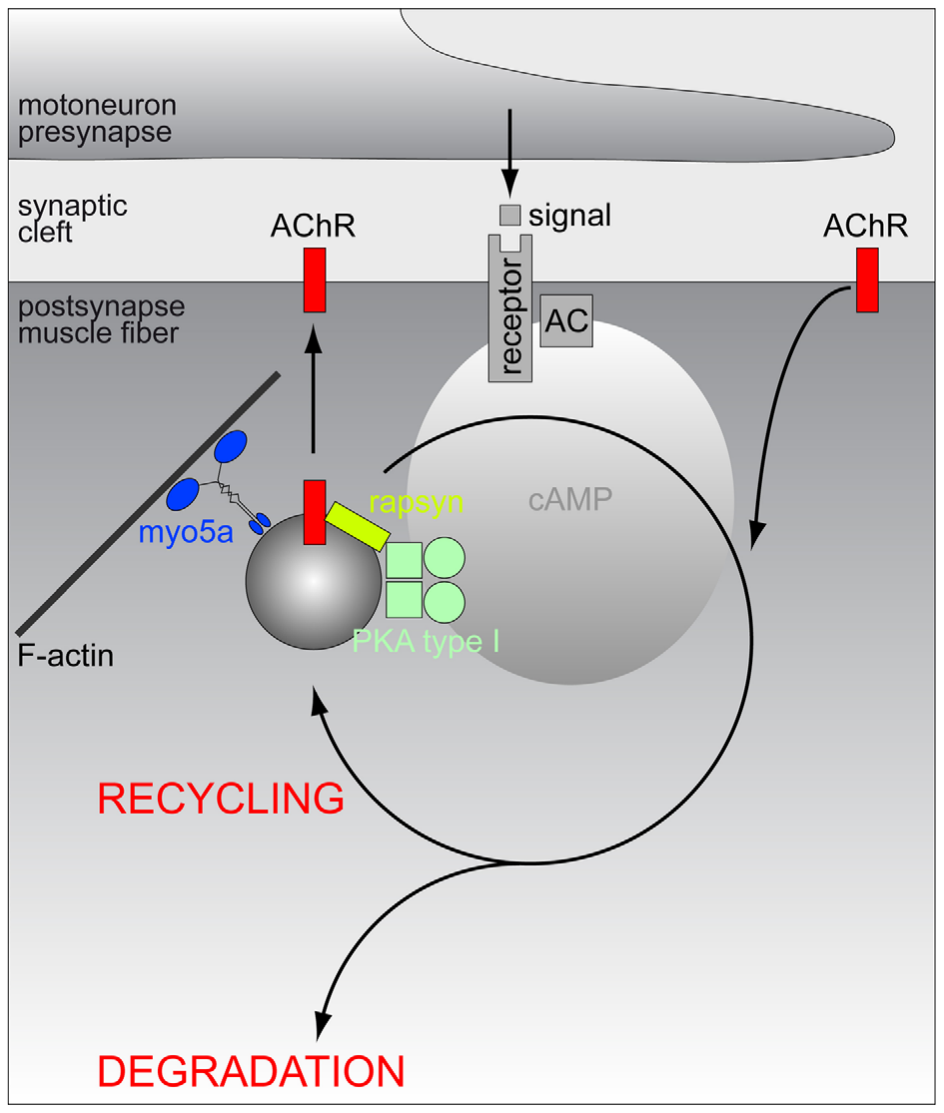
**Schematic model of the hypothetic assembly of a PKA microdomain beneath the NMJ**. Amongst other potential roles at the NMJ, PKA-RI is critical for proper lifetime regulation of AChR. At least a part of these regulatory processes appears to be linked to the endocytosis/recycling of AChR. From the endocytic compartment, AChRs may either return to the postsynaptic membrane (recycling) or be routed to a degradation pathway. To carry out its function, PKA-RI needs to be recruited to endocytic/recycling vesicles which transport AChR and those vesicles are to be tethered close to the postsynaptic membrane in order to receive local rises of cAMP levels. Anchoring of PKA-RI to these vesicles is mediated by rapsyn, while myo5a serves to restrain endocytic/recycling vesicles in the actin-rich cortex underneath the NMJ. First messengers triggering the relevant local rises in cAMP levels are still elusive and they might originate from motoneurons, sympathetic nervous system or other sites.

As for the metabolic stabilization of AChRs different lines of evidence indicate the involvement of the neuropeptide, α-calcitonin-gene related peptide (αCGRP), and of cAMP/PKA-dependent pathways (Poyner, 1992). αCGRP was found to raise postsynaptic cAMP levels in the PKA-RIα microdomain (Röder et al., 2010) and to rapidly phosphorylate the α- and δ-subunit of AChR (Miles et al., 1987, 1989). Furthermore, αCGRP treatment changed the electrophysiological characteristics of AChR (Mulle et al., 1988) and it rescued denervation-induced fragmentation of NMJs (Röder et al., 2010). Furthermore, αCGRP was described to counteract PKC-induced destabilization of AChRs (Li et al., 2001, 2002) and to stimulate AChR gene expression (New and Mudge, 1986; Fontaine et al., 1987) as well as synaptic strength (Lu et al., 1993). Experiments using AKAP disruptor peptides suggested that the proper localization of PKA-RIα on the aforementioned subsynaptic puncta is essential for AChR stabilization (Röder et al., 2010) and *in vivo* imaging and biochemical assays revealed that many of these structures indeed contain endocytosed AChR (Röder et al., 2010). Altogether these findings suggest that the PKA-RIα positive puncta represent αCGRP-sensitive PKA microdomains on endocytic carriers containing AChR. The actin-dependent motor protein, myosin Va, was found to be crucial for tethering these carriers in close proximity to the NMJ (Röder et al., 2010) (for schematic, see Figure 1), but which is the AKAP used for anchoring PKA-RIα to the AChR-laden carriers? Previous reports suggested D-AKAP1 (Barradeau et al., 2001, 2002; Perkins et al., 2001) as a candidate. However, this was purely based on the general enrichment of this protein underneath the NMJ. A recent study followed another rationale and looked for a protein that would (1) target to AChR, (2) exhibit an AKAP-typical α-helical coiled-coil domain and (3) have interaction domains with other signaling components, and, based on these pre-requisites, tested the hypothesis that rapsyn serves as AKAP at this place. Rapsyn is a 43 kDa protein, that was originally co-purified with AChR from Torpedo electroplax and that quantitatively and strongly interacts with AChR (Sobel et al., 1977; Neubig et al., 1979; Porter and Froehner, 1985; Froehner, 1993). From N- to C-terminus, rapsyn contains a myristoylation site, seven tetratricopeptide repeats, an amphipathic α-helical region, a RING-domain, and PKA- and PKC-phosphorylation consensus sites (Ramarao and Cohen, 1998; Ramarao et al., 2001). Notably, full-length rapsyn but not rapsyn lacking its α-helical domain co-precipitated with PKA-RIα (Choi et al., 2012). *In silico* modeling identified functional sequence homology of that region with PKA-interaction domains of different AKAPs, and rapsyn interacted with PKA-RIα in a bimolecular fluorescence complementation assay both, in cells and *in vivo* (here in subsynaptic puncta) (Choi et al., 2012). Finally, over-expression of a peptide derived from the rapsyn α-helical coiled-coil domain displaced PKA-RIα from the NMJ puncta and severely impaired AChR stability (Choi et al., 2012), strongly arguing for rapsyn as the AKAP responsible for linking PKA-RIα to the subsynaptic PKA microdomain (for schematic, see Figure 1).

NMJ structure and stability of AChR strongly suffer in muscles from the *mdx* mouse model for Duchenne muscular dystrophy (DMD) (Torres and Duchen, 1987; Lyons and Slater, 1991; Xu and Salpeter, 1997; Grady et al., 2000; Shiao et al., 2004), in which cAMP signaling is aberrant (Reynolds et al., 2008). That does, of course, not mean that the altered signaling is the underlying cause for the NMJ phenomena, but the typical subsynaptic enrichment of PKA-RIα was lacking in about half of all fibers in *mdx* muscles, microdomain specificity to different GPCR agonists was subverted, and AChR turnover was inversely correlated with PKA-RIα accumulation beneath the NMJ (Röder et al., 2012). Altogether this set of data suggests a link between defect subsynaptic microdomain formation of PKA-RIα in dystrophic muscles and the observed alterations in NMJ morphology and AChR stability. In general, the concept of an AChR stabilizing role of cAMP was also tested in the context of another devastating group of muscle diseases, i.e., congenital myasthenic syndromes (CMS). These are rare genetic diseases that affect either pre- or postsynaptic components of the NMJ and lead to impaired neuromuscular transmission and muscle weakness (Palace and Beeson, 2008). Many forms of CMS also present low levels of AChR at the NMJ. Although the underlying mechanisms for that might differ between distinct mutations, the finding that sympathomimetic substances, such as ephedrine and salbutamol, can significantly improve these patients' symptoms (Edgeworth, 1930; Schara et al., 2009; Lashley et al., 2010; Liewluck et al., 2011; Finlayson et al., 2013), suggests an involvement of catecholamines in AChR turnover. Since ephedrine and salbutamol both can activate β_2_-ARs and thus affect cAMP production, this could point to a possible role of cAMP in stabilizing AChR and/or leading to higher AChR expression. Certainly, further research is needed to better understand these effects.

### PKA microdomains at the sarcomeric region

**Figure 2:**
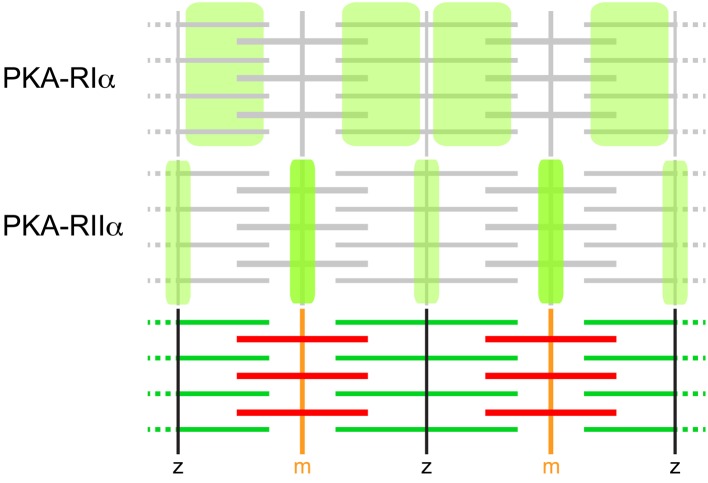
**PKA-R isoforms display differential distribution patterns in skeletal muscle sarcomeres**. Immunohistochemical and GFP-based sensor analyses showed that PKA-RIα essentially co-distributes with microfilaments and PKA-RIIα with m-bands and z-lines. These distribution patterns are the most prevalent in mouse muscle. They are based on AKAP-binding and can be subverted in diseased muscle, such as upon dystrophy. AKAPs relevant for these distribution patterns are still elusive, but myospryn is very likely to participate in the anchoring of PKA-RIIα.

Sarcomeric PKA microdomain organization was addressed either by immunohistochemical staining of PKA-R isoforms (Perkins et al., 2001) or by expression studies using fluorescent protein-labeled Epac-based cAMP biosensors (Nikolaev et al., 2004) targeted to PKA microdomains by virtue of D/D domains (Di Benedetto et al., 2008) specific for either PKA-RIα (RIα-EPAC-camps) or PKA-RIIα (RIIα-EPAC-camps) (Röder et al., 2009). Both approaches yielded essentially identical results (for schematic, see Figure 2). While PKA-RIα was found in a broad band overlapping with the sarcomeric actin filaments, PKA-RIIα exhibited highly confined striated localization that coincided with both, the m-band and the z-line. Experiments using over-expression of AKAP disruptor peptides showed that this peculiar distribution pattern is largely based on AKAP-dependent subcellular targeting (Röder et al., 2009). Harnessing the cAMP-sensor domain of the EPAC-camps biosensors furthermore showed a differential sensitivity of the two microdomains. While the cAMP concentration in the RIα-microdomain was elevated in the presence of the agonist, αCGRP, the RIIα-microdomain responded to norepinephrine with increased cAMP levels (Röder et al., 2009). Both effects were ablated by AKAP disruptor peptides (Röder et al., 2009). These data demonstrate that the sarcomeric region of skeletal muscle exhibits clearly defined and functionally distinct PKA microdomains, which are organized by specific AKAPs. At present, it is unclear which AKAP(s) mediate the anchoring of PKA-RIα and PKA-RIIα to the different domains in the sarcomeric region but one eminent protein, myospryn, is certainly carrying out a part of this function. This 449 kDa heavy protein with the official gene name CMYA5 (cardiomyopathy-associated 5) was identified by expression profiling of a cardiac muscle library and has since been found to interact specifically with PKA-RIIα but not (or hardly) with the other PKA-R isoforms (Reynolds et al., 2007). Intriguingly, endogenous myospryn localization in the sarcomere exhibited the expected m- and z-line expression pattern fitting to PKA-RIIα distribution (Reynolds et al., 2007) while in another study myospryn showed only faint m-line and strong I-band distribution (Sarparanta et al., 2010). Whether this could indicate natural variability or be due to other factors is unclear, but myospryn is now widely considered to be an important determinant for PKA microdomain formation in skeletal and heart muscle. In the recent past, more and more proteins were found to interact with myospryn, including the structural proteins α-actinin (Durham et al., 2006), desmin (Kouloumenta et al., 2007), dystrophin (Reynolds et al., 2008), and titin (Sarparanta et al., 2010), as well as proteolytic enzymes such as the muscle-specific protease, calpain 3 (Sarparanta et al., 2010), and the protein phosphatase calcineurin (Kielbasa et al., 2011). Notably, these proteins all play important roles in muscle integrity and metabolic adaptations suggesting a mediator role of myospryn in these processes (Sarparanta, 2008). This is corroborated by feedback loops: Expression of myospryn is modulated by the cAMP-dependent CREB pathway, and it is known to be a direct target of the myocyte enhancer factor MEF2A (Durham et al., 2006). Furthermore, absence or malfunction of myospryn is observed in a couple of muscle diseases including tibial and limb-girdle muscular dystrophies (TMD and LGMD2J, respectively) (Sarparanta et al., 2010) as well as the most abundant and severe form of muscular dystrophies, i.e., DMD (Reynolds et al., 2008). Notably, in the DMD mouse model, *mdx*, myospryn showed altered subcellular distribution and specific PKA activity was strongly reduced (Reynolds et al., 2008). This also fits to another study, where PKA-RIα distribution in the sarcomeres was altered and, in particular, the microdomain selectivity to respond to the specific agonists, norepinephrine and αCGRP, was completely subverted (Röder et al., 2009). In summary, although the precise function of cAMP microdomain organization in skeletal muscle sarcomeres is still elusive, there are correlations between aberrant cAMP signaling and severe muscle diseases. Based on this rationale, urocortins were tested as therapeutics against muscular dystrophy (Hinkle et al., 2007; Reutenauer-Patte et al., 2012). Urocortins are neuropeptides that bind to the GPCRs, corticotropin-releasing factor (CRF) receptors (CRFR), of which CRF_2_R is highly abundant in skeletal muscle. Notably, in dystrophic *mdx* mice treatment with urocortins significantly ameliorated a set of symptoms, ranging from fiber necrosis to muscle function. Possible mechanisms of action might include cAMP-induced activation of PKA and Epac, which in turn may address altered Ca^2+^ handling in skeletal muscle fibers (Reutenauer-Patte et al., 2012).

## Mechanisms of cAMP-induced effects on skeletal muscle protein metabolism

Skeletal muscle constitutes about 40–60% of our body masses. It is, thus, not only driving locomotion but also represents a major metabolic organ due to its enormous energy expenditure, its capability to take up glucose in an insulin-dependent manner, and its role as amino acid-source during catabolic conditions (Sandri, 2008; Glass, 2010). All these functions are intimately linked to the sarcomeres, which constitute the vast excess of skeletal muscle tissue. GPCR- and cAMP-mediated signaling can act on different time scales, ranging from the seconds to days range, correlating to either direct activation of targets (e.g., by PKA-dependent phosphorylation) or to changes in transcriptional profiles (e.g., by modulation of CREB activity). In contrast to their catabolic effects on lipids and carbohydrate metabolism, catecholamines exert an anabolic effect on skeletal muscle protein metabolism (Navegantes et al., 2002). This effect is mediated by β_2_-ARs and involves cAMP signaling (Navegantes et al., 2000, 2002). Numerous studies have shown that β_2_-adrenergic agonists, such as clenbuterol (“older” generation) and formoterol (“newer” generation), induce hypertrophy of skeletal muscle in rodents, large animals and humans (Lynch and Ryall, 2008). β-agonist-induced hypertrophy seems to be specific for striated muscle, since smooth muscles do not increase in size in response to these agents (Reeds et al., 1986) and β2-adrenergic agonists inhibit smooth muscle cell proliferation (Southgate and Newby, 1990; Tomlinson et al., 1994; Indolfi et al., 1997). Experiments conducted in β_2_-AR^−/−^ mice (Hinkle et al., 2002) have convincingly shown that β_2_-AR is responsible for this anabolic effect. Indeed, β_2_-AR^−/−^ mice display decreased cross-sectional area of type I and IIA fibers compared with age-matched wildtype mice (Bacurau et al., 2009), an effect that is associated with lower muscle cAMP levels (Gonçalves et al., 2009).

**Figure 3:**
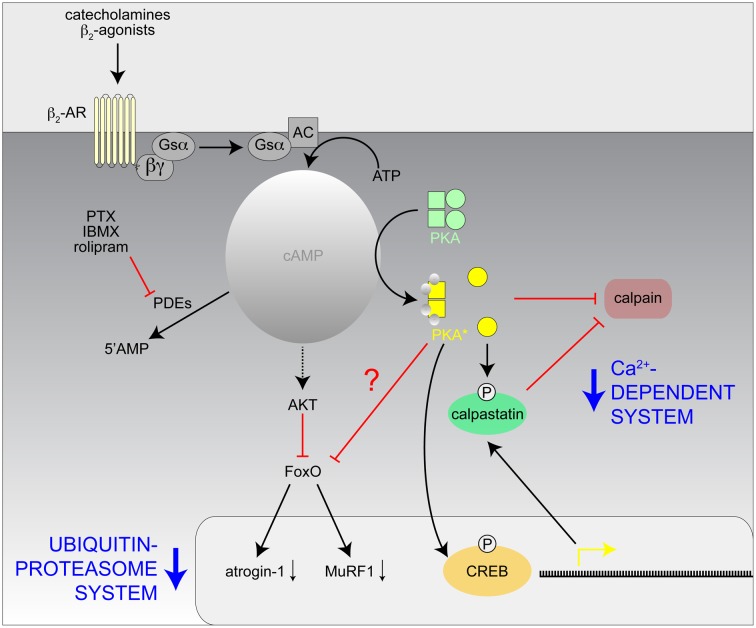
**Hypothetical model of the mechanisms involved with the inhibition of the Ca^2+^-dependent and Ubiquitin-proteasome proteolytic systems in skeletal muscle by catecholamines and β_2_-agonists**. AC, adenylate cyclase; CREB, cAMP response element binding protein; IBMX, isobutylmethylxanthine; PKA^*^, activated PKA; PTX, pentoxifylline; ?, unknown effect.

The molecular mechanisms by which cAMP signaling induces growth and muscle-sparing responses are uncertain and may involve an increase in the rate of protein synthesis and/or a decrease in protein degradation (Navegantes et al., 2002; Lynch and Ryall, 2008). A large body of evidence indicates that the *in vivo* effects of cAMP-inducing agents are in part due to inhibition of muscle proteolysis (Figure 3). Indeed, both chemical and surgical sympathectomy in fed rats lead to an increase in the activity of the Ca^2+^-dependent proteolytic system, which suggests the existence of an adrenergic tonus on skeletal muscle that keeps this pathway inhibited under normal conditions (Navegantes et al., 1999, 2001). Accordingly, the administration of β_2_-adrenergic agonists is accompanied by a reduction in calpain 1 activity and an increase in the activity of calpastatin, an endogenous inhibitor of calpains (Bardsley et al., 1992; Parr et al., 1992). More recently, it has been demonstrated that β_2_-adrenergic agonists might attenuate muscle atrophy through inhibitory effects on the ubiquitin-proteasome system, the main intracellular pathway for protein degradation in skeletal muscle (Yimlamai et al., 2005; Gonçalves et al., 2012). This effect is mediated through a cAMP/Akt-dependent pathway (Kline et al., 2007; Gonçalves et al., 2009, 2012), which leads to the phosphorylation of Foxo3a and, consequently, the suppression of atrogin-1/MAFbx and MuRF1, two ubiquitin E3-ligases involved in muscle atrophy (Bodine et al., 2001; Centner et al., 2001; Lecker et al., 2004; Sandri et al., 2004). Moreover, treatment with PDE inhibitors increased muscle cAMP levels and decreased the rate of total protein degradation in muscles from diabetic (Baviera et al., 2007) and fasted rodents (Lira et al., 2007) through a clear reduction in the activity of the Ca^2+^-dependent proteolytic system and the ubiquitin-proteasome system. The fact that the antiproteolytic effect of both β_2_ agonists (Gonçalves et al., 2012) and PDE inhibitors (Baviera et al., 2007) *in vitro* was inhibited by H89, a PKA inhibitor, and mimicked by 6-BNZ-cAMP, a PKA activator, further supports the idea that activation of the cAMP cascade via a PKA-dependent pathway is one of the regulatory mechanism(s) to prevent excessive skeletal muscle protein breakdown. Given that in dystrophic muscle the Ca^2+^-dependent proteolytic system and the ubiquitin-proteasomal system are activated on the one hand (Kar and Pearson, 1976; Spencer and Tidball, 1996; Kumamoto et al., 2000) and PKA signaling, on the other hand, is disturbed (Reynolds et al., 2008; Röder et al., 2009), it is reasonable to suggest that increased calpain and proteasome activities contribute to dystrophic pathology and, by extension, that protease inhibition by cAMP-inducing agents could be a treatment strategy for DMD.

## On the origin and destination of catecholamines in skeletal muscle

**Figure 4:**
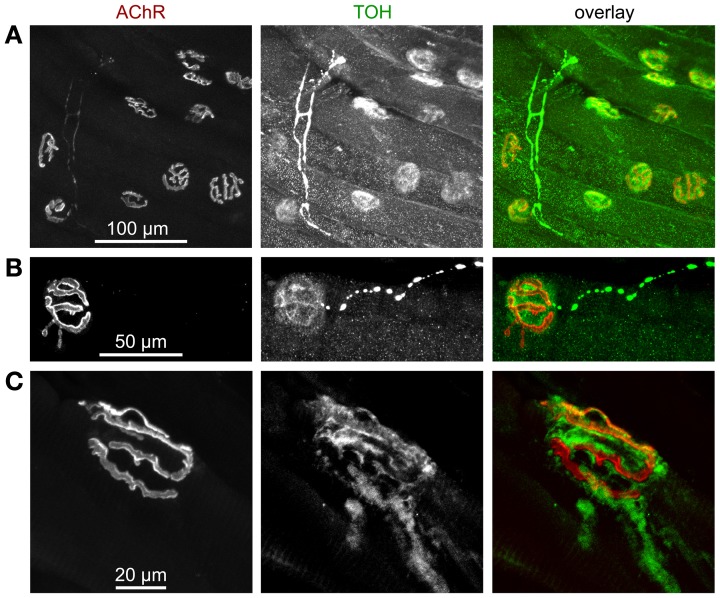
**Tyrosine hydroxylase (TOH) immunofluorescence is present in sparse axon-like processes and at NMJs**. Mouse hindlimb muscles were sectioned and then stained with α-bungarotoxin-AlexaFluor555 (AChR) and an antibody against TOH. Then, confocal microscopy was performed. All panels show maximum z-projections of several optical slices. From left to right, fluorescence signals of AChR, TOH, and overlays are depicted. In overlays, AChR and TOH appear in red and green, respectively. **(A)** Overview picture showing that most NMJs display enrichments of TOH immunofluorescence. **(B)** Note thin and pearl chain-like TOH-positive process that ends next to TOH-positive accumulation, which shows a complementary distribution with respect to AChR. **(C)** Detail of a NMJ with TOH staining complementary to AChR labelling and with emanating axon-like process.

It is general knowledge that sympathetic first messengers can be released from either adrenal medulla as hormones or from sympathetic neurons as neurotransmitters directly onto target tissues (Mason, 1968). However, surprisingly little is known about the real contributions of these different modes of sympathetic activities in most tissues (Daly and McGrath, 2011) and this holds true also for skeletal muscle. Yet, to our knowledge, there are a few studies reporting on direct innervation of skeletal muscle fibers by non-myelinated, noradrenergic fibers (Boeke, 1909a,b, 1913; Barker and Saito, 1981; Tadaki et al., 1995), suggesting that sympathetic actions on skeletal muscle are at least partially mediated by neural mechanisms. Accordingly, a study using surgical ablation of sympathetic ganglia, which innervate hind limb muscles have shown that direct innervation of skeletal muscles by autonomic nerves is critical for muscle homeostasis (Navegantes et al., 2004) and a wealth of investigations has dealt with the effects of sympathetic agonists on skeletal muscle force potentiation and release of acetylcholine from motoneurons (see, e.g., Oliver and Schäfer, 1895; Goffart and Ritchie, 1952; Krnjevic and Miledi, 1958; Bowman and Raper, 1967). The latter are processes, which are likely to need fast regulation in the course of fight-or-flight situations. This triggered us to reinvestigate the distribution of sympathetic innervation in skeletal muscle and to address differences between sympathetic targets in healthy and dystrophic muscles. Thus, we first studied the distribution of the sympathetic neuron marker, tyrosine hydroxylase (TOH) in longitudinal sections of mouse hindlimb muscle and found this marker protein to be concentrated on top of most NMJs (Figure 4A). This is in accordance with previous studies carried out in several vertebrate species, including man (Chan-Palay et al., 1982a,b). In many cases, enrichments of TOH immunostaining in proximity to NMJs were connected to pearl chain-like processes, which are likely to represent sympathetic axons (Figure 4B). Notably, while previous investigators performed immunostainings on transverse muscle sections and thus proposed TOH to be present in the motoneuronal presynaptic portion of NMJs (Chan-Palay et al., 1982a,b), the analysis of our longitudinal slices revealed that TOH immunofluorescence does not match postsynaptic AChR staining as it would be typical for motoneuronal markers, but was mostly just in the gaps between the NMJ pretzel structure (Figure 4C). That fits to the older observations from Boeke based on tissue silver impregnation (Boeke, 1909a,b, 1913) and corroborates his suggestion that sympathetic neurons run and terminate next to motoneurons. Future investigations should be carried out to further strengthen this finding.

**Figure 5:**
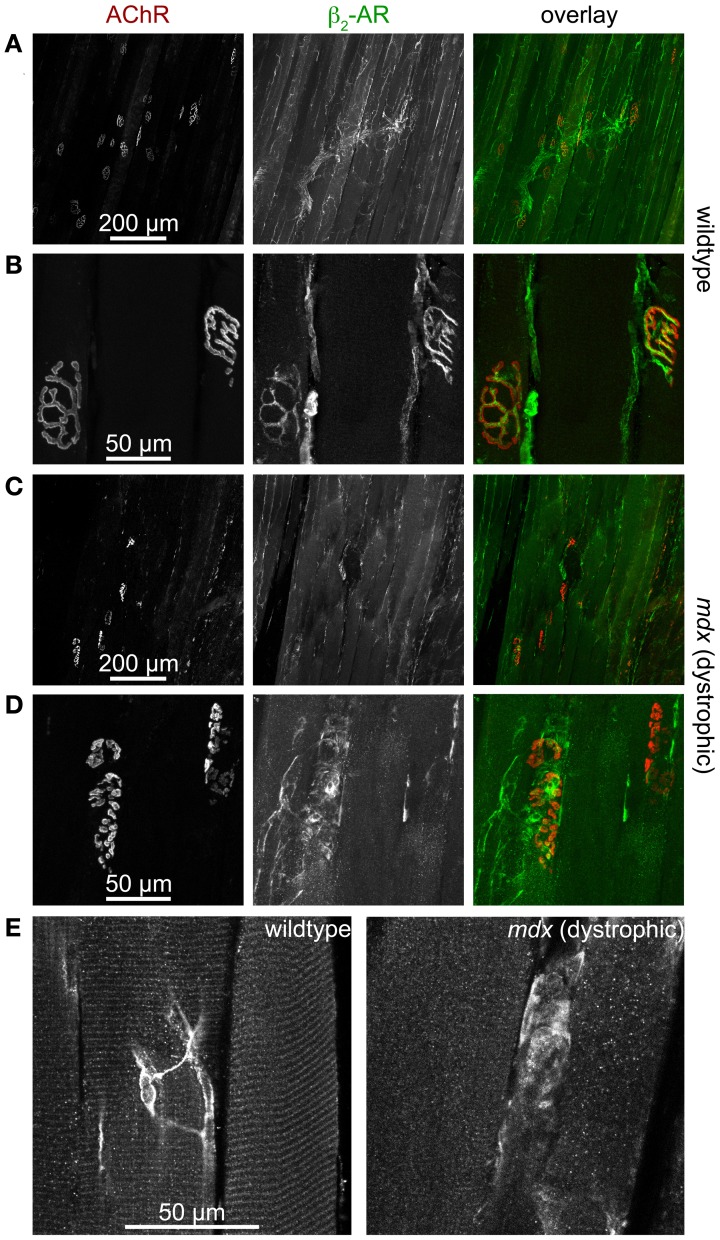
**β_2_-AR-immunofluorescence is found in motoneurons and muscle fibers and is severely altered in dystrophic muscle**. Mouse hindlimb muscles of wildtype (**A,B,E** left) or dystrophic mdx mice (**C,D,E** right) were sectioned and then stained with α-bungarotoxin-AlexaFluor555 (AChR) and an antibody against β_2_-AR. Then, confocal microscopy was performed. **(A–D)** Show maximum z-projections of several optical slices, in **(E)** single optical slices are depicted. From left to right, fluorescence signals of AChR, β_2_-AR, and overlays are depicted. In overlays, AChR and β_2_-AR appear in red and green, respectively. In wildtype muscles, β_2_-AR immunofluorescence covers entire motor nerve bundles **(A)** and perfectly matches the AChR arborized structures in the NMJ **(B)**. This is typical for the distribution of motoneuronal markers. Conversely, β_2_-AR immunofluorescence is much sparser in dystrophic muscle **(C)** and exhibits only partial overlap with AChR staining **(D)**. In muscle fibers of wildtype animals **(E** left**)** β_2_-AR is found in triple striations per sarcomer, similar to the distribution of PKA-RIIα (see Figure 2). This striation is mostly absent in dystrophic muscle **(E** right**)**, where β_2_-AR distribution is often uniform along the fibers. Finally, anostomotic β_2_-AR-positive, axon-like processes of unknown identity are also often seen running along muscle fibers **(E** left**)**.

Next, we addressed the expression pattern of β_2_-AR in hindlimb muscle. This showed immunohistochemical signals of β_2_-AR in at least four different locations: (1) larger blood vessels (not depicted), (2) motoneurons (Figures 5A,B), (3) muscle fibers (Figure 5E, left panel), and (4) ill-defined anastomotic fibers (Figure 5E, on left panel see central part of the picture). Since the presence of β_2_-AR had been found by staining and anticipated to be present due to functional roles in blood vessels (Daly and McGrath, 2011), motoneurons (Melamed et al., 1976; Wohlberg et al., 1986; Bondok et al., 1988; Adachi et al., 1992; Parkis et al., 1995; Zeman et al., 2004; Tartas et al., 2010; Noga et al., 2011; Baker and Baker, 2012) and muscle fibers (Gross et al., 1976; Cairns and Dulhunty, 1993a,b; Cairns et al., 1993; Kokate et al., 1993; Navegantes et al., 1999, 2000, 2001, 2002, 2003, 2004; Prakash et al., 1999; Decostre et al., 2000; Gonçalves et al., 2012), our findings in wildtype muscles were corroborating previous reports. However, the difference between wildtype and dystrophic *mdx* muscles was striking, both with respect to neuronal as well as muscle staining: First, while the typical pretzel-shaped postsynaptic AChR signals in wildtype muscle were perfectly mirrored by presynaptic β_2_-AR staining (Figures 5A,B) in almost fibers, this was much rarer the case in *mdx* synapses (Figures 5C,D), which were also highly fragmented as reported previously (Torres and Duchen, 1987; Lyons and Slater, 1991; Grady et al., 2000). Second, while β_2_-AR immunofluorescence displayed a highly regular striated patterning in wildtype muscle (Figure 5E, left panel), it was almost uniformly distributed in many fibers from *mdx* muscles (Figure 5E, right panel). In summary, these data show that there are significant differences in distribution of β_2_-AR between healthy and dystrophic muscles. In the context of the PKA microdomain hypothesis this could be an additional level of dysregulation leading to alterations of cAMP with all the sequelae as discussed before.

### Conflict of interest statement

The authors declare that the research was conducted in the absence of any commercial or financial relationships that could be construed as a potential conflict of interest.
